# Practical management of new oral anticoagulants after total hip or total knee arthroplasty

**DOI:** 10.1007/s12306-013-0306-8

**Published:** 2013-11-19

**Authors:** W. Klauser, M. Dütsch

**Affiliations:** 1 Orthopaedic Department, Helios ENDO Klinik Hamburg, Holstenstraße 2, 22767 Hamburg, Germany; 2 Helios ENDO Klinik, Hamburg, Germany

**Keywords:** Oral anticoagulants, Deep vein thrombosis, Hip and knee arthroplasty

## Abstract

Within the past 5 years, the oral anticoagulants rivaroxaban, apixaban, and dabigatran etexilate have been approved for the prevention of venous thromboembolism in adult patients after elective hip or knee arthroplasty in the European Union and many other countries worldwide. These agents differ from the previously available anticoagulants because they selectively and directly inhibit a single factor in the coagulation cascade—rivaroxaban and apixaban inhibit Factor Xa, and dabigatran inhibits Factor IIa (thrombin)—potentially enhancing the predictability of their anticoagulant effect. Currently, although some guidelines provide recommendations for the use of rivaroxaban, dabigatran etexilate, and apixaban in clinical practice, there are still questions regarding the optimal practical management of patients receiving these agents. This article briefly reviews the practical limitations associated with conventional anticoagulants, discusses potential issues with the practical management of the newer oral anticoagulants, and provides clinical experience from a single institution where rivaroxaban and dabigatran etexilate have been used within their approved indications.

## Introduction

Venous thromboembolism (VTE), which comprises deep vein thrombosis and pulmonary embolism, is a potentially life-threatening complication of major orthopedic surgery. Unfractionated heparin (UFH), low molecular weight heparins (LMWHs), and vitamin K antagonists (VKAs) have been used for the prevention of VTE after major orthopedic surgery for more than 20 years [1, 20]. In Europe, the LMWH enoxaparin has become the standard of care in this indication. The practical management of these anticoagulants is well established, and their limitations have been well documented; these include parenteral administration in the case of UFH and the LMWHs, and unpredictable pharmacology in the case of VKAs [3, 33].

**Fig. 1:**
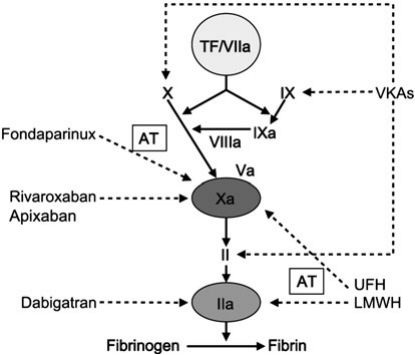
The coagulation cascade and anticoagulant targets. *a* activated, *AT* antithrombin, *LMWH* low molecular weight heparin, *TF* tissue factor, *UFH* unfractionated heparin, *VKA* vitamin K antagonist

In 2005, the synthetic pentasaccharide fondaparinux was approved in the European Union (EU) for the prevention of VTE after major orthopedic surgery. Fondaparinux indirectly inhibits Factor Xa, an enzyme of the coagulation cascade involved in the generation of thrombin, via the cofactor antithrombin [66]. The mechanism of action of fondaparinux differs from conventional anticoagulants in that it selectively targets a single factor in the coagulation cascade (Fig. 1), increasing the predictability of its anticoagulant effect in comparison with UFH and the VKAs [13]. However, use of fondaparinux is still limited by the fact that it is administered by subcutaneous injection. In 2008, two direct oral anticoagulants were approved in the EU for the prevention of VTE in adult patients after elective hip or knee arthroplasty: the direct Factor Xa inhibitor rivaroxaban and the direct thrombin inhibitor dabigatran (the active form of dabigatran etexilate). In 2011, a third oral anticoagulant—the direct Factor Xa inhibitor apixaban—was approved in the same indication. Like fondaparinux, these three agents selectively inhibit a single target in the coagulation cascade, but unlike fondaparinux, they are not dependent on binding to antithrombin for their activity (Fig. 1) [52, 61, 69].

The internationally recognized American College of Chest Physicians (ACCP) guidelines for the prevention of VTE recommend anticoagulation with LMWH, fondaparinux, dabigatran, apixaban, rivaroxaban, low-dose UFH, or a VKA after total hip arthroplasty (THA) or total knee arthroplasty (TKA) [20]. Thromboprophylaxis is recommended for a minimum of 10–14 days after THA or TKA; however, it is suggested that extending treatment for up to 35 days may provide additional benefit [20]. The UK National Institute for Health and Clinical Excellence (NICE) and the Scottish Medicines Consortium recommend the use of rivaroxaban, dabigatran, or apixaban within their marketing authorization, as alternatives to established agents for the prevention of VTE after elective THA or TKA [47–49, 57–59]. The Association of the Scientific Medical Societies in Germany also makes recommendations for dabigatran and rivaroxaban in this indication [4].

Rivaroxaban and dabigatran have now been available for the prevention of VTE after elective hip or knee arthroplasty for more than 4 years in the EU, while apixaban was approved for the same indication in May 2011. This review focuses on the clinical experience with rivaroxaban and dabigatran in a single institution and discusses potential issues surrounding the practical management of these agents.

## Established anticoagulants: practical management issues

As a result of their long-established use, conventional anticoagulants are well trusted. However, their effective management necessitates a number of practical considerations. In Europe, LMWHs are recommended to be given preoperatively [20], which prevents same-day hospital admission for elective surgery, and can complicate decisions related to the method of anesthesia. In Europe, UFH is usually administered subcutaneously at a low dose; LMWHs and fondaparinux are also administered subcutaneously. This can make their long-term use inconvenient, particularly after hospital discharge. If it is necessary to administer high-dose UFH intravenously, the variable anticoagulant response that patients have to UFH necessitates dose adjustment and routine coagulation monitoring [33]. Furthermore, use of UFH is known to have a risk of heparin-induced thrombocytopenia [28]. Because of these limitations, the use of UFH has largely been replaced by LMWHs, although these drugs have their own limitations [28].

In Germany, it is considered good clinical practice to administer LMWH for a period of 28 days after THA or TKA. However, because the median length of hospital stay for THA or TKA outside the USA is 10 days [24], patients will often be discharged after surgery with injections that require self-administration; in the USA, the median length of hospital stay is even shorter (3 days after THA and 4 days after TKA) [24]. Many patients do not like self-injecting, and others, especially elderly patients, may require assistance with injections. A family member may need to be trained to give the injections, or nurse visits may be necessary. As a result, many patients stop taking LMWH when they have left the hospital, placing themselves at increased risk of VTE. In an observational study conducted in six general hospitals and six rehabilitation centers in Germany, 71.9 % of 178 patients said they would prefer oral rather than subcutaneous administration of VTE prophylaxis. Furthermore, a time saving of 46.13 s was observed in tablet preparation and administration compared with syringe preparation and administration [50].

When parenteral thromboprophylaxis is used at home, there can be self-administration issues. In telephone interviews conducted in the Netherlands in 687 patients who had undergone THA or TKA, a total of 511 (74.4 %) patients indicated that they used parenteral thromboprophylaxis at home. However, 48.8 % of these patients reported administration problems, including pain, bruising, and itching. Home-care visits for parenteral administration problems were required by 9.9 % (95 % confidence interval 6.4–13.4) and 9.6 % (95 % confidence interval 5.8–13.4) of THA and TKA patients, respectively. Approximately 60 % of all THA or TKA patients said they would prefer oral rather than parenteral thromboprophylaxis [11].

LMWHs are predominantly excreted renally, and in patients with impaired renal function, accumulation can occur, potentially increasing the risk of bleeding. Renal clearance is also the primary mode of elimination for fondaparinux [28]. In the Global Orthopaedic Registry (GLORY), the median age of patients undergoing THA or TKA was over 65 years [24]. Because renal function is known to decrease with age [30], the use and/or dose of LMWHs or fondaparinux should be considered carefully in this patient population. The ACCP guidelines recommend that in patients with renal impairment, the use of anticoagulants that accumulate should be avoided. Alternatively, a lower drug dose should be used, or drug levels and anticoagulant effect should be monitored [29].

VKAs have the advantage of being administered orally. However, because of other factors including genetics, considerable variability in dose response is observed among patients. For this reason, VKAs require regular coagulation monitoring and dose adjustments to ensure that patients stay within the therapeutic range [target international normalized ratio (INR) of 2.5 (range 2.0–3.0)] to reduce the risk of adverse events [1]. VKAs also have numerous food and drug interactions [1].

To minimize the risk of bleeding when anticoagulants are used together with epidural catheters, guidelines on the timing of catheter placement and withdrawal, in relation to dosing and peak plasma concentrations, are available for UFH, LMWHs, and VKAs [2, 34]. All patients receiving anticoagulants, including fondaparinux, with regional anesthesia and postoperative indwelling epidural catheters should be closely monitored for signs and symptoms of neurological impairment [2, 31, 34].

If an overdose occurs, UFH can be fully reversed using intravenous protamine sulfate. LMWHs do not have a specific antidote, although the effects can be partially reversed using protamine sulfate [28]. In the event of a VKA overdose, vitamin K administered orally or intravenously can be used, although its full effects can take up to 24 h [5]. For more rapid reversal, vitamin K may need to be supplemented with factor concentrates [3]. Fondaparinux has no known antidote [31].

## Management of direct oral anticoagulants

### Timing of first dose

The optimal timing of the first dose of an anticoagulant, in order to achieve a good balance between efficacy and safety, has always been a controversial issue. Evidence from a number of anticoagulant clinical trials of varying size and robustness has been reviewed [51]. The findings, predominantly from the trials of LMWHs, suggest that efficacy is not dependent on preoperative anticoagulant initiation and that 6 h is the threshold for early postoperative administration. When the first dose was initiated within 6 h postoperatively, the risk of major bleeding increased without improved efficacy, whereas initiation 6 h postoperatively was effective but not associated with an increased risk of major bleeding. However, initiation 12–24 h postoperatively could be less effective than initiation 6 h postoperatively [51].

Unlike LMWHs, rivaroxaban, apixaban, and dabigatran are initiated postoperatively [7, 10, 12, 20], allowing the convenience of hospital admission on the day of surgery. For the prevention of VTE after elective hip or knee arthroplasty, rivaroxaban 10 mg is administered once daily as a single tablet. It is recommended that the first dose of rivaroxaban is administered 6–10 h postoperatively if adequate hemostasis has been established [7]. Apixaban is initiated 12–24 h after surgery at a dose of 2.5 mg twice daily. Initiation within this time window is at the physician’s discretion, depending on the need to balance the benefit of earlier VTE prophylaxis with the risk of postoperative bleeding [12]. Dabigatran is administered as a once-daily 220 mg dose, taken as two capsules. The recommendation is that the first dose is administered 1–4 h after surgery as a half-dose (110 mg), continuing with two capsules once daily thereafter. If adequate hemostasis is not achieved, initiation of therapy should be delayed [10]. All patients are monitored intensely the night after surgery to check blood pressure and infusions. Therefore, if the first dose of oral anticoagulant is due during the night, dosing should not be delayed.

In our clinic, patients receiving general anesthesia during THA or TKA are administered the first dose of rivaroxaban 8–10 h postoperatively. In patients receiving regional anesthesia with an epidural catheter, the first dose of rivaroxaban is administered after the same postoperative time period. In the case of dabigatran, the first dose is administered 4–6 h postoperatively after general anesthesia. In patients receiving regional anesthesia with an epidural catheter, dabigatran is started after removal of the catheter.

### Vomiting

One consideration when using oral rather than parenteral anticoagulants is the possibility of postoperative vomiting and the impact this may have on the effectiveness of the drug. A number of different factors could increase the risk of postoperative vomiting, including certain anesthetics, perioperative use of analgesics such as opioids, intense preoperative anxiety, or a history of migraine [27]. Rivaroxaban, dabigatran, and apixaban all have a fast onset of action. Rivaroxaban reaches peak plasma concentrations between 2 and 4 h after administration [7] and apixaban between 3 and 4 h after administration [12]. Dabigatran reaches peak plasma concentrations within 2 h of administration [10]. A fast onset of action is an advantage for an oral anticoagulant because the period when postoperative vomiting might be considered to be an issue, i.e., before the drug is absorbed, is relatively short. If a patient vomits more than 4 h after rivaroxaban or apixaban administration, or more than 2 h after dabigatran administration, no additional anticoagulation should be necessary. In clinical practice, if a patient receiving rivaroxaban or apixaban vomits within 4 h and the tablet is seen in the vomit, the dose is taken again if possible. If it is not clear whether the tablet has been regurgitated or not, the next tablet of rivaroxaban is taken 24 h after the last tablet intake. A similar procedure is followed if vomiting occurs soon after administration of dabigatran. Given its twice-daily dosing, the next apixaban tablet is taken 12 h after the last tablet intake. Nausea and vomiting can be managed with the use of antiemetics and antinauseants as required.

In our clinic, nausea and vomiting have not so far proved an issue when using the newer oral anticoagulants. If vomiting occurs, it is usually within the first 4 h after surgery, before administration of the first dose, or the following morning when the patient is mobilized for the first time. In the latter case, the first dose has been administered many hours previously, and the next dose is administered at the normal time.

### Concomitant medications

As previously highlighted, patients undergoing THA or TKA are often elderly and may already be taking one or more medications. In addition, nonsteroidal anti-inflammatory drugs and acetylsalicylic acid, which are known to affect bleeding risk, may be prescribed for pain relief after major orthopedic surgery [22, 23, 32]. It is, therefore, important that any direct oral anticoagulant does not interact with these drugs or other commonly used medications. Based on interaction studies, the recommendations for the use of rivaroxaban, apixaban, and dabigatran with other drugs are summarized in Table 1. Although it has been shown that these drugs do not have clinically relevant interactions with nonsteroidal anti-inflammatory drugs or acetylsalicylic acid [12, 15, 19, 36], because of the risk of hemorrhage, patients should always be observed closely for signs of bleeding when using these drugs concomitantly.

**Table 1:** Interaction of comedications with rivaroxaban, dabigatran, and apixaban

Anticoagulant	Interaction/recommendation	Medication
Rivaroxaban	No clinically relevant interaction	Platelet aggregation inhibitors, including acetylsalicylic acid [15, 36] Nonsteroidal anti-inflammatory drugs [15] Naproxen [7] Enoxaparin [7] Digoxin [7] Atorvastatin [7] Ranitidine [41] Clopidogrel [37]
	Use with caution [7]	Fluconazole Strong CYP3A4 inducers
	Not recommended [7]	Systemic azole antimycotics HIV protease inhibitors Strong inhibitors of both CYP3A4 and P-gp Dronedarone
Dabigatran	No clinically relevant interaction	Acetylsalicylic acid [19] Nonsteroidal anti-inflammatory drugs [19] Diclofenac [10] Digoxin [10] Atorvastatin [10]
	Dose reduction recommended [10]	Amiodarone
	Use with caution [10]	Strong P-gp inhibitors, e.g., verapamil or clarithromycin Quinidine
	Not recommended [10]	Potent P-gp inducers, e.g., rifampicin Unfractionated heparins and heparin derivatives Low molecular weight heparins Fondaparinux Desirudin Thrombolytic agents GPIIb/IIIa receptor antagonists Clopidogrel Ticlopidine Dextran Sulfinpyrazone Vitamin K antagonists
Apixaban	No clinically relevant interaction	Acetylsalicylic acid [12] Digoxin [25] Inhibitors or substrates of CYP enzymes [68] Naproxen [12] Atenolol [12] Famotidine [12]
	Use with caution [12]	Strong inducers of both CYP3A4 and P-gp, e.g., rifampicin, phenytoin, carbamazepine, phenobarbital Nonsteroidal anti-inflammatory drugs
	Not recommended [12]	Strong inhibitors of both CYP3A4 and P-gp, e.g., ketoconazole, ritonavir Unfractionated heparins and heparin derivatives Low molecular weight heparins Fondaparinux Desirudin Thrombolytic agents GPIIb/IIIa receptor antagonists Clopidogrel Dipyridamole Dextran Sulfinpyrazone Vitamin K antagonists Other oral anticoagulants

*CYP* cytochrome P450, *HIV* human immunodeficiency virus, *P*-*gp* P-glycoprotein

### Coagulation monitoring

In clinical trials, rivaroxaban, apixaban, and dabigatran were found to have predictable pharmacokinetics and pharmacodynamics [26, 38–40, 63, 64]. Because of their predictable anticoagulant effects, none of these agents requires routine coagulation monitoring. However, although coagulation monitoring is not required, there may be circumstances in the clinic when it is useful to determine the pharmacodynamic activity of these agents, for example, in the event of an overdose or during a hemostatic emergency. Several clotting assays have been evaluated for this purpose [21, 56]. There is evidence that the INR, specifically designed for monitoring VKAs, is not suitable for monitoring rivaroxaban [60]. Prothrombin time (PT) assays calibrated to rivaroxaban concentrations may be useful to qualitatively assess the plasma levels of rivaroxaban if required, but results should be expressed as rivaroxaban concentration (ng/mL) and a central PT reagent should be used to minimize variability [54]. An anti-Factor Xa chromogenic assay has also been shown to provide quantitative measurements of rivaroxaban plasma concentrations [55]. However, these assays are not yet routinely available. For apixaban, measuring inhibition of Factor Xa activity using an anti-Factor Xa assay gives a better indication of plasma apixaban concentrations compared with the PT test and was shown to detect apixaban even at low plasma concentrations [6, 8]. Options for measuring the anticoagulant activity of dabigatran include the ecarin clotting time assay, and the commercially available hemoclot thrombin inhibitor assay (HYPHEN BioMed, Andresy, France) developed by the manufacturers of dabigatran [62, 63].

### Antidote

In the event of an overdose, or during a rapidly progressing hemostatic emergency, such as a gastrointestinal or intracranial bleeding event, strategies for the reversal of the anticoagulant effect must be considered. Like LMWHs and fondaparinux, none of the direct oral anticoagulants has specific antidotes that can fully reverse their effects. However, in clinical practice, specific reversal of anticoagulants with relatively short half-lives, such as rivaroxaban, apixaban, and dabigatran, is not usually necessary. Rivaroxaban has a mean terminal half-life of 5–9 h in young individuals and 11–13 h in the elderly [7]; dabigatran has a mean terminal half-life of 12–14 h in healthy young individuals and 11 h in healthy elderly individuals [10]. The mean terminal half-life of apixaban is approximately 12 h [12].

In the event of an overdose, or in a bleeding emergency, a number of steps can be taken to reverse the effects of rivaroxaban. Activated charcoal can be used to reduce absorption. If bleeding occurs, discontinuation or delay of the next dose is recommended. If bleeding continues, an appropriate symptomatic treatment, such as blood product or component transfusion, should be considered. Administration of a specific procoagulant reversal agent, such as prothrombin complex concentrate (PCC), activated PCC (aPCC), or recombinant Factor VIIa, should be considered if previous measures are unsuccessful; however, there is limited clinical experience with these products in patients receiving rivaroxaban [7]. Both aPCC (FEIBA^®^ Baxter AG, Vienna, Austria) and PCC (Kanokad^®^, Courtaboeuf, France; Cofact, Sanquin Blood Supply, Amsterdam, the Netherlands) have shown potential for the reversal of rivaroxaban in ex vivo studies in healthy volunteers, but further investigation is required [14, 46].

The use of oral activated charcoal has been shown to reduce apixaban exposure in healthy subjects [12]. If there are bleeding complications, treatment should be discontinued and bleeding treated using standard methods. As with rivaroxaban, treatment with recombinant Factor VIIa can be considered if bleeding cannot be controlled; however, there is currently no clinical experience with this treatment for patients receiving apixaban.

In the event of a dabigatran overdose, the following steps should be taken: Treatment should be discontinued and the source of bleeding investigated; because dabigatran is excreted predominantly by the renal route, adequate diuresis must be maintained; initiation of the appropriate treatment, for example, surgical hemostasis or the transfusion of fresh frozen plasma, should be considered. Dabigatran can be dialyzed, but the clinical utility of this approach has not been determined [10]. In an ex vivo study, PCC was unable to reverse the anticoagulant effect of dabigatran; however, particle reversal was achieved with recombinant Factor VIIa and aPCC [14, 46].

### Use of catheters

**Fig. 2:**
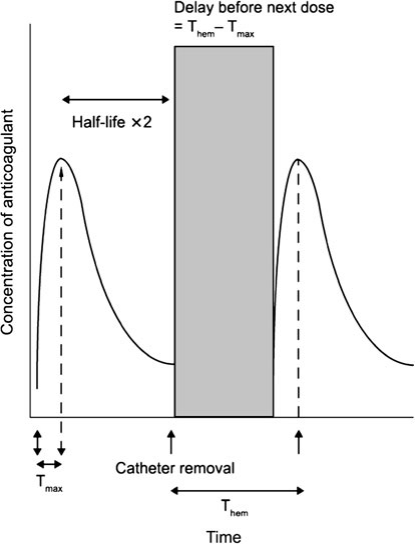
Regional anesthesia management strategy. It is recommended that for a specific anticoagulant, catheter removal should occur after at least two half-lives have elapsed. The next dose of anticoagulant should not be administered until time to reach hemostasis (*T*_hem_) minus time to reach maximum plasma concentration (*T*_max_) has elapsed (*T*_hem_ − *T*_max_)

A consideration when using any anticoagulant together with regional anesthesia or spinal/epidural puncture is the increased risk of epidural or spinal hematoma. A recommended management strategy to reduce the risk of hemorrhagic events associated with regional anesthesia in patients receiving newer anticoagulants has been proposed [53]. This strategy is based on the pharmacokinetic properties of the specific anticoagulant, including the time required to reach maximal concentration, the half-life, and the dose regimen (Fig. 2). It is recommended that epidural catheters should not be removed until at least two half-lives after the last injection of the anticoagulant [53].

Based on this proposal, and the pharmacokinetic profile of rivaroxaban, it is recommended that an epidural catheter should not be removed earlier than 18 h after the last administration of the drug. The next rivaroxaban dose should be administered no earlier than 6 h after the removal of the catheter, and if traumatic puncture occurs, rivaroxaban administration should be delayed for 24 h [7]. Epidural catheters should be removed at least 5 h before initiating apixaban treatment [12]. For patients with indwelling catheters, it is recommended that 20–30 h should elapse between the last dose of apixaban and catheter removal. The use of dabigatran is not recommended in anesthetized patients with postoperative indwelling epidural catheters. The first dose of dabigatran should be administered at least 2 h after the catheter is removed, and these patients should be observed frequently for neurological signs and symptoms [10].

### Adverse drug reactions

Three phase III trials compared rivaroxaban 10 mg once daily with the EU-approved dose of enoxaparin (40 mg once daily) for the prevention of VTE after THA (RECORD1 and RECORD2) or TKA (RECORD3). RECORD1 and RECORD3 were head-to-head drug regimen comparisons, whereas RECORD2 compared extended-duration rivaroxaban with short-duration enoxaparin followed by placebo [16, 35, 42]. A fourth trial compared rivaroxaban 10 mg once daily with enoxaparin 30 mg twice daily after TKA [67]. Treatment-emergent, drug-related adverse events occurring in 1–10 % of any treatment group in these four trials were nausea, postprocedural hemorrhage, fever, peripheral edema, and increase in transaminases [7].

Two phase III studies compared apixaban 2.5 mg twice daily with the EU-approved enoxaparin dose for the prevention of VTE after TKA (ADVANCE-2) or THA (ADVANCE-3) [44, 45]. A third trial compared apixaban 2.5 mg twice daily with enoxaparin 30 mg twice daily after TKA [43]. Treatment-emergent adverse events that occurred in 1–10 % of patients in clinical studies of apixaban were anemia, hemorrhage, nausea, and contusion [12].

Two phase III trials compared two doses of dabigatran (150 or 220 mg once daily) with the EU-approved enoxaparin regimen for the prevention of VTE after THA (RE-NOVATE) or TKA (RE-MODEL) [17, 18]. Dabigatran was compared with enoxaparin 30 mg twice daily in another phase III trial in TKA patients [65]. In these clinical trials, adverse events occurring in 1–10 % for both doses were anemia, hemoglobin decrease, epistaxis, gastrointestinal hemorrhage, abdominal pain, diarrhea, dyspepsia, and nausea [10].

In clinical practice in this institution, serious side effects have not been observed with rivaroxaban or dabigatran, although elevated transaminases and gamma-glutamyl transpeptidases leading to drug discontinuation have been observed more frequently with dabigatran. Anecdotally, in the wider orthopedic community, these direct oral anticoagulants have sometimes been associated with higher rates of postoperative effusions or bleeding compared with LMWH. It has been routine clinical practice to administer LMWH preoperatively, and in some cases, this practice has continued even after the introduction of the direct oral anticoagulants. The omission of preoperative LMWH resulted in a substantial decrease in these side effects. This highlights the importance of education for surgeons and anesthetists about how these drugs work and the potential implications of mismanagement.

### Contraindications

As with all drugs, the direct oral anticoagulants are contraindicated for use in certain patient groups. Rivaroxaban, dabigatran, and apixaban are contraindicated in patients who are hypersensitive to the active substances or to any of the excipients; have clinically significant active bleeding; have lesions or a condition that puts them at significant risk of major bleeding; or are receiving concomitant treatment with any other anticoagulant [7, 10, 12]. Apixaban and rivaroxaban are also contraindicated in patients with hepatic disease associated with coagulopathy and risk of clinically relevant bleeding, and these agents should not be used in patients who are pregnant or breast-feeding [7, 12]. Dabigatran is also contraindicated in patients who have severe renal impairment (creatinine clearance <30 mL/min); have hepatic impairment or liver disease expected to have any impact on survival; have prosthetic heart valves requiring anticoagulant treatment; or are receiving concomitant treatment with strong P-glycoprotein inhibitors [10].

## Conclusion

The ACCP guidelines include recommendations on the use of rivaroxaban, dabigatran, and apixaban for the prevention of VTE after THA or TKA [20]. Clinical experience with these newer oral agents is relatively limited, especially for apixaban, but they appear to be well tolerated and easily managed in the clinic. Potential issues with their management, such as the effect of postoperative vomiting on efficacy, or the reversal of the anticoagulant effect in an emergency, can generally be managed with routine clinical procedures. The direct oral anticoagulants possess many advantages over the more established agents, including administration of the first dose postoperatively, no requirement for routine coagulation monitoring, and limited drug–drug interactions. Furthermore, the convenience of oral administration could improve patient compliance postdischarge, although, as with any anticoagulant that is self-administered, effective patient education in this area is crucial [9]. Clinical experience with rivaroxaban, dabigatran, and apixaban so far supports the potential of these drugs to improve and simplify routine management of VTE prevention postoperatively.
